# Oligomerization of SCF^TIR1^ Is Essential for Aux/IAA Degradation and Auxin Signaling in Arabidopsis

**DOI:** 10.1371/journal.pgen.1006301

**Published:** 2016-09-12

**Authors:** Mohammad H. Dezfulian, Espanta Jalili, Don Karl A. Roberto, Britney L. Moss, Kerry Khoo, Jennifer L. Nemhauser, William L. Crosby

**Affiliations:** 1 Department of Biological Sciences, University of Windsor, Windsor, Ontario, Canada; 2 Department of Biology, University of Washington, Seattle, Washington, United States of America; National University of Singapore and Temasek Life Sciences Laboratory, SINGAPORE

## Abstract

The phytohormone auxin is a key regulator of plant growth and development. Molecular studies in Arabidopsis have shown that auxin perception and signaling is mediated via TIR1/AFB–Aux/IAA co-receptors that assemble as part of the SCF^TIR1/AFB^ E3 ubiquitin-ligase complex and direct the auxin-regulated degradation of Aux/IAA transcriptional repressors. Despite the importance of auxin signaling, little is known about the functional regulation of the TIR1/AFB receptor family. Here we show that TIR1 can oligomerize *in planta* via a set of spatially clustered amino acid residues. While none of the residues identified reside in the interaction interface of the TIR1-Aux/IAA degron, they nonetheless regulate the binding of TIR1 to Aux/IAA substrate proteins and their subsequent degradation *in vivo* as an essential aspect of auxin signaling. We propose oligomerization of TIR1 as a novel regulatory mechanism in the regulation of auxin-mediated plant patterning and development.

## Author Summary

The phytohormone auxin plays a diverse and critical role in plant growth and development. In Arabidopsis, the F-box protein TIR1 is a component of an E3 SCF ubiquitin-ligase complex that serves as the auxin receptor and directs the ubiquitin-dependent degradation of repressors of auxin signaling known as the Aux/IAA proteins. Little is known regarding the stoichiometry of the SKP1, CUL1, RBX1 and TIR1 subunits that comprise the SCF^TIR1^ complex. Here, we show that TIR1 is capable of oligomerization *in planta*. We also show that mutant alleles that abolish TIR1 oligomerization impair the degradation of known SCF^TIR1^ substrates and fail to restore auxin signaling and response in *tir1* loss-of-function genetic backgrounds. The results suggest that TIR1 homo-oligomerization is an important aspect of the regulation of SCF^TIR1^ function and auxin signaling.

## Introduction

Indole-3-acetic acid (auxin) is a small tryptophan-derived phytohormone and a key regulator of diverse aspects of plant growth and development [1, 2]. A family of six Auxin-signaling F-box binding proteins (TIR1/AFBs), together with Auxin/INDOLE-3-ACETIC ACID (Aux/IAA) proteins are known to act as the internal co-receptors for auxin binding and activation of an SCF^TIR1/AFB^ E3 ubiquitin (Ub)-ligase complex [3–7]. Aux/IAA proteins act as negative regulators of auxin signaling through their interaction with Auxin Response Factors (ARFs) [5, 8, 9]. The Aux/IAA transcription repressors have been biochemically characterized as the prime targets for SCF^TIR1/AFB^ E3 Ub-ligase complexes in the presence of auxin molecules [5, 6, 8]. Consequently, in the presence of auxin, the abundance of Aux/IAA proteins is reduced and downstream ARF response genes are activated as part of the generalized auxin response [5, 6]. Notwithstanding the central role of auxin perception to plant patterning and development, a number of key questions remain to be addressed. Whereas the dynamic regulation of TIR1/AFB receptor proteins is anticipated to be central to auxin perception, most proposed SCF ligase-related auxin regulatory mechanisms have been described at the level of the CUL1 subunit involving neddylation, CAND1 binding, and de-neddylation via the COP9 signalosome [10–12]. Hence, these modifications can be considered as part of the general regulation of SCF ligase homeostasis, functioning independent of the AFB auxin receptors. An exception is that of TIR1 *S*-nitrosylation, which was shown to regulate TIR1-Aux/IAA interaction thus providing a means by which nitric oxide (NO) can modulate auxin signaling. However, the underlying molecular mechanism regulating TIR1-Aux/IAA interaction via this redox-based modification in the absence of auxin is not clear [13]. As well, the finding that neither of the two residues proposed to be sites of S-nitrosylation reside at the TIR1-Aux/IAA interaction interface complicates a structure-function interpretation of the results. Indeed, others have shown that additional residues outside of the Aux/IAA degron interface contribute to either the binding or the degradation of these proteins via an unknown mechanism [4, 14, 15]. Additionally, a mechanistic framework has yet to be described for how a SCF^TIR1/AFB^ achieves the spatial flexibility required to efficiently ubiquitinate such a diverse set of proposed Aux/IAA substrate proteins. Taken together, it is likely that additional uncharacterized regulatory mechanisms important for auxin signal perception in plants remain to be elucidated. This suggestion is supported by reports of multiple independent missense alleles of TIR1 that affect auxin signaling, albeit via unknown mechanisms [16, 17]. Strikingly, several of these residues (C140, G147 and D170) are spatially clustered, suggesting that the molecular basis of the observed phenotypes may involve a common structural basis.

The formation of higher order SCF ligase structures can provide a means for regulation of their ubiquitination activity [18]. However, the structure-function implications associated with the formation of higher order E3 SCF ligase structures remain poorly understood and are controversial [18, 19]. Two working models have been forwarded in this regard: the first proposes that SCF ligase dimerization stimulates ubiquitin conjugation while having little effect on the affinity for its substrate protein [20]. A second model argues that dimerization increases affinity for substrates proteins via interaction with multiple degron domains [21, 22]. Overall, the current perspectives lack a comprehensive framework for understanding the contribution of individual SCF ligase subunit components to the formation of higher-order structures.

An assessment of the oligomerization potential of SCF^TIR1/AFB^ could provide a novel mechanistic approach for understanding the receptor-based regulation of auxin perception and downstream signaling. To this end, we have investigated the formation of higher-order quaternary structures involving TIR1 as the major intercellular auxin receptor in Arabidopsis, with implications for auxin response and regulation. Our results reveal a novel aspect of auxin signaling regulation at the receptor level and provide a framework within which diverse aspects of TIR1 structure and function can be integrated.

## Results

### TIR1 protein lacks a canonical F-box dimerization-domain

Select members of the SCF subclass of E3 Ub-ligases can dimerize and this dimerization is mediated through a conserved domain (D-domain) located immediately N-terminal of the F-box domain in participating F-box subunits [20–22]. We have undertaken a bioinformatics survey among AFB proteins in Arabidopsis, but failed to identify the presence of a canonical D-domain within this family of proteins. The result suggests that, if TIR1 protein does indeed oligomerize, the oligomerization is mediated via a novel dimerization domain.

It has been suggested that the absence of a canonical D-domain in some F-box proteins correlates with the formation of a monomeric SCF ligase complexes [18]. Recent reports have indicated that human Skp2 and Fbx4 both persist in monomeric form *in vitro* yet can oligomerize *in vivo* following acetylation and phosphorylation, respectively [23–25]. We undertook to examine the potential for SCF^TIR1^ dimerization *in vivo* using the heterologous *Nicotiana benthamiana* leaf transient expression system, having first demonstrated the fidelity of the Nicotiana expression system for the *in vivo* recapitulation of TIR1-dependent Aux/IAA protein degradation inherent to the Arabidopsis auxin-signaling pathway (**S1 Fig**).

**Fig 1:**
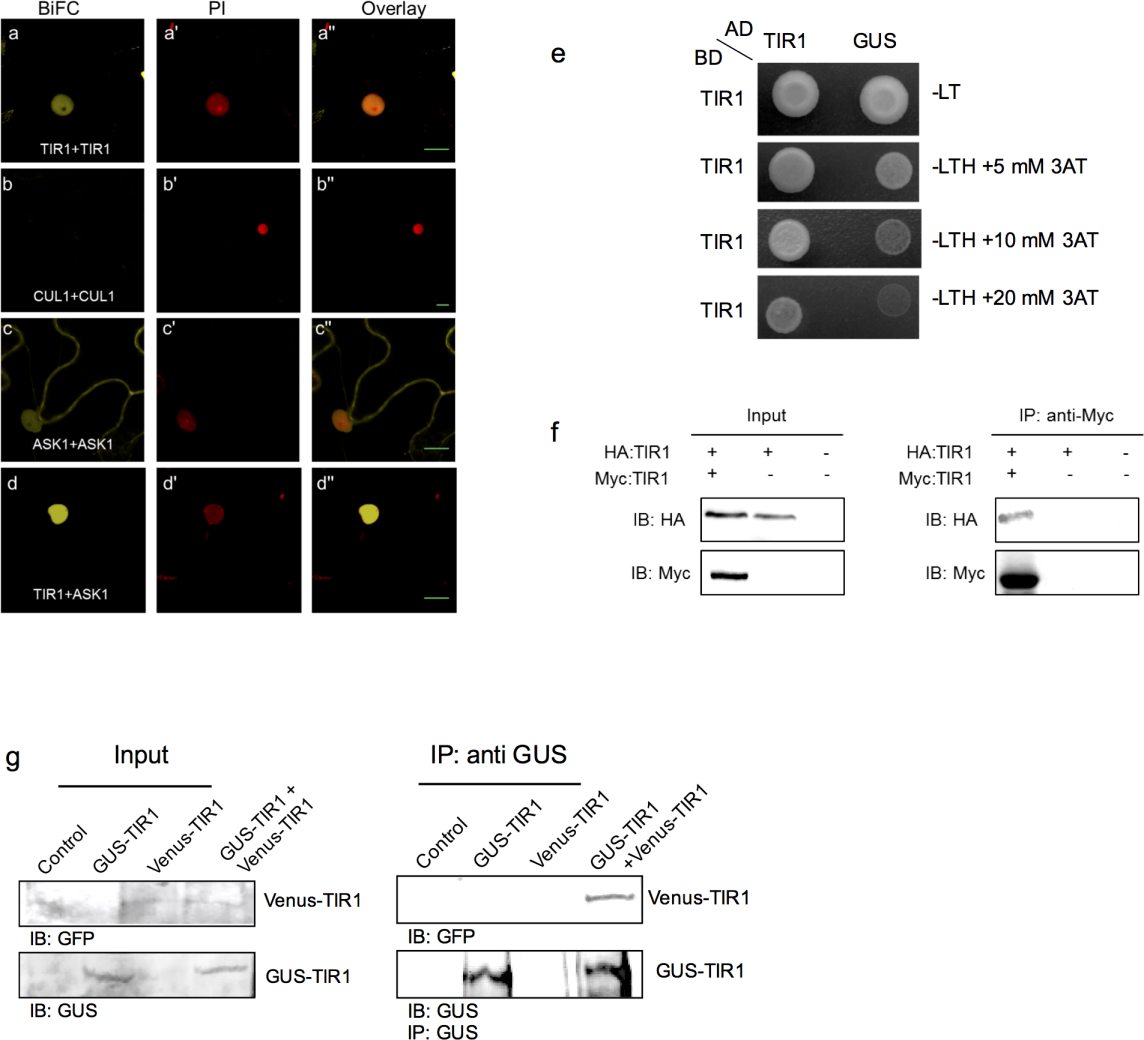
Evaluation of oligomerization potential of SCF^TIR1^ subunits. **(a-d)** BiFC-based evaluation of TIR1, CUL1, ASK1 oligomerization and ASK1-TIR1 interaction, respectively, following transient expression in Nicotiana leaves. **(a'-d')** Propidium iodide staining of the nucleus. **(e)** Assessment of TIR1-TIR1 protein interaction in a Y2H assay. Images of single colonies expressing the designated constructs and grown on histidine plates (top panel) and test plates containing the indicated concentrations of 3-AT in the absence of histidine (bottom panels). **(f)** Co-IP-based assessment of TIR1 oligomerization following transient expression in Nicotiana leaves. HA:TIR1 and Myc:TIR1 were co-injected in leaves. Protein extracts were subjected to immunoprecipitation using anti-Myc antibody. Immunoprecipitates were examined by western-blotting using anti-Myc and anti-HA antibodies. (**g**) Co-IP-based assessment of TIR1 oligomerization in double *pTIR1*:*TIR1–GUS* and *pTIR1*:*gTIR1–VENUS* transgenic Arabidopsis plants. Protein extracts were subjected to immunoprecipitation using anti-GUS antibody. Immunoprecipitates were examined by western-blotting using anti-GUS and anti-GFP antibodies.

We used multiple complimentary approaches to assess TIR1 oligomerization. Initially, a bimolecular fluorescence complementation (BiFC) approach was used to assess the *in vivo* oligomerization of three known subunits of the SCF^TIR1^ complex. Fluorescence signals arising from oligomerization between TIR1 and ARABIDOPSIS SKP1-LIKE1 (ASK1) were observed, but not for CUL1-CUL1 (**Fig 1A–1C**). The sub-cellular localization pattern of the TIR1-TIR1 interaction is reminiscent of that observed for the TIR1-ASK1 interaction (**Fig 1D**). The inability to detect CUL1 oligomerization in our system is most probably due to limitations associated with the BiFC technique, given that we were similarly unable to detect a CUL1-ASK1 interaction using this approach. The protein interactions observed using BiFC were validated using the yeast two-hybrid (Y2H) system as well as co-immunoprecipitation (Co-IP) in Nicotiana (**Fig 1E and 1F**). In addition, we assessed the oligomerization of TIR1 in Arabidopsis in transgenic lines expressing TIR1-GUS and TIR1-VENUS fusion proteins in a *tir1-1* mutant background. Both the *pTIR1*:*TIR1-GUS* and *pTIR1*:*gTIR1-VENUS* constructs express TIR1 behind its native promoter and have been shown to complement the auxin response phenotypes observed in *tir1-1* plants [26, 27]. Using Co-IP-based experiments involving extracts prepared from plants expressing these TIR1 fusions we assessed the oligomerization of TIR1 by GUS antibody to enrich for the TIR1–GUS fusion protein, and subsequently analyzing for the enrichment of TIR1-VENUS. The results confirmed the *in vivo* oligomerization of TIR1 in Arabidopsis **(Fig 1G)**, notwithstanding the absence of a canonical F-box dimerization domain.

### TIR1 oligomerization is independent of Aux/IAA binding

**Fig 2:**
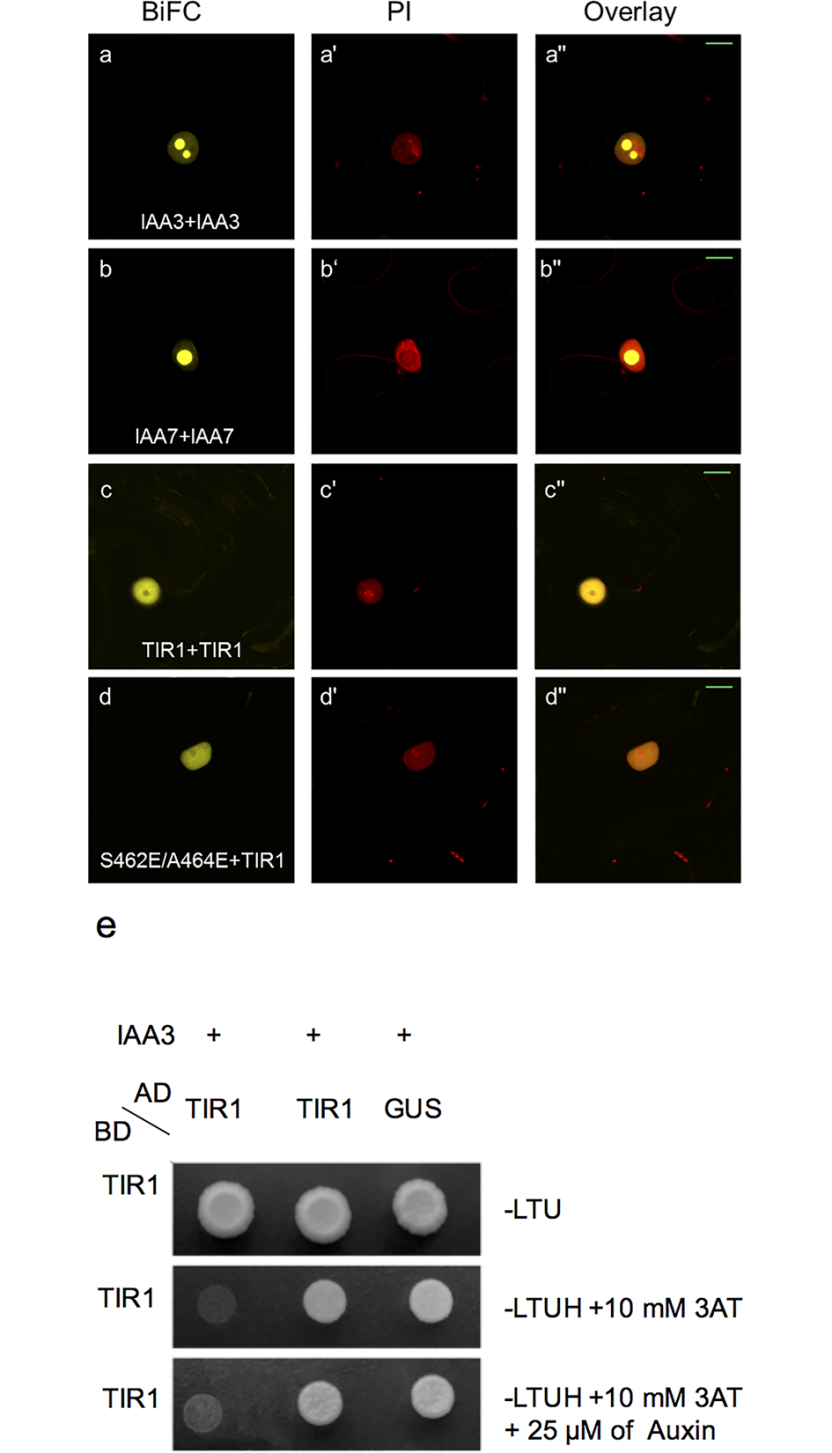
TIR1 oligomerization is independent of Aux/IAA binding. **(a** and **b)** BiFC-based assessment of IAA3 and IAA7 oligomerization respectively. **(c)** BiFC-based assessment of wild-type TIR1 interaction with S462E/A464E-TIR1. **(d)** BiFC-based assessment of TIR1 oligomerization. **(e)** Assessment of TIR1-TIR1 protein interaction in a Y2H assay in the presence and absence of Aux/IAA3 and 25 μM of Indole-3-acetic acid (auxin). Images of single colonies expressing the indicated constructs and grown on histidine plates (top panel) and test plates containing 10 mM of 3-AT (middle panel) and test plates simultaneously containing 10 mM of 3-AT and 25 μM of Indole-3-acetic acid (bottom panel).

Arabidopsis Aux/IAA proteins have been shown to form hetero- or homo-dimers consistent with their role as transcriptional regulators [28–30]. We investigated the possibility that oligomerization of Aux/IAA proteins could in turn lead to the oligomerization of TIR1 *in planta* when in complex with the SCF ligase. Accordingly, we assessed the oligomerization of IAA7 and IAA3 using the Nicotiana BiFC system where both proteins were found to oligomerize within the nucleus (**Fig 2A and 2B).** However, the localization pattern of the oligomerization was distinct from that observed for TIR1 oligomerization in that the signal was confined to discrete regions within the nucleus. We examined the localization of YFP-IAA protein fusions and found that both IAA3 and IAA7 exhibited similar localization patterns to that of the BiFC interaction. The differential localization pattern of TIR1 versus IAA oligomerization suggests that the oligomerization of each pair is spatially and likely functionally independent.

Structural studies have identified a number of key residues that participate in TIR1-Aux/IAA assembly [4, 7]. These residues can be classified into three groups based on their domain-localization in TIR1: those that are directly in contact with Aux/IAA proteins, those resident in the auxin binding pocket, and those located at the inositol-1,2,3,4,5,6- hexabisphosphate (InsP_6_) cofactor-binding pocket. We evaluated the oligomerization potential of the S462E/A464E-*tir1* double mutant allele, which was previously shown to disrupt the TIR1-Aux/IAA degron interaction [7]. This mutant retained the ability to oligomerize in the BiFC system **(Fig 2D)**. Auxin has been shown to enhance TIR1-Aux/IAA protein affinities by extending the protein-interaction interface between the two proteins; its presence might be expected to act as a “molecular glue” facilitating protein-protein interactions [7]. Thus, missense alleles that abolish auxin binding would be expected to correspondingly abolish Aux/IAA binding. To this end, we generated three mutants (L439A-*tir1*, R403A-*tir1* and S438A/L439A-*tir1*) wherein residues in contact with auxin were mutated. All three mutant alleles were found to retain the ability to oligomerize (**S2A–S2C Fig**). In TIR1, the InsP_6_ binding pocket is flanked by positively charged residues that are thought to provide the foundation for auxin binding [7], Thus, abolishing InsP_6_ interaction would be expected to disrupt TIR1 interaction with Aux/IAA proteins. To evaluate this possibility, we generated a mutant wherein two InsP_6_-contacting residues (K113, K114) were mutated to alanine. This double mutant was likewise found to retain the ability to oligomerize in the BiFC system (**S2D Fig**). To further explore the potential role of Aux/IAA co-expression on TIR1 oligomerization we performed Y2H experiments in the presence and absence of auxin and Aux/IAA3 protein. The Y2H data indicated that co-expression of Aux/IAA3 and treatment with auxin did not detectably affect the level of TIR1 oligomerization (**Fig 2E**). Although it cannot be excluded that unidentified proteins may contribute to or mediate TIR1 oligomerization *in planta*, the experimental evidence obtained here, combined with Y2H data, suggests that TIR1 oligomerization is independent of Aux/IAA oligomerization. This result may be significant in light of previously published data showing that the abundance of Aux/IAA homo-dimers were markedly reduced in TIR1 co-transfected samples [31].

### ASK1 stabilizes TIR1 protein via masking of the hydrophobic F-box domain

We assessed the potential role of ASK1 in TIR1 oligomerization by generating a variant allele harboring a deletion in the degenerate F-box domain (TIR1-ΔF(Δ10–40)) and assessed its interaction with both ASK1 and full-length TIR1 in the BiFC system. Interestingly, TIR1-ΔF did not detectably interact with either ASK1 (**S3B Fig**) or full-length TIR1 (**S3A Fig**), suggesting that ASK1 may be essential for TIR1 oligomerization. The inability of TIR1-ΔF to oligomerize could be due to an altered tertiary structure resulting in aberrant protein folding or to decreased stability. To investigate this, we generated two alleles containing amino acid substitution mutations within the F-box domain of TIR1, V33E-TIR1 and V33A/K35A-TIR1. When the variant constructs were expressed and submitted to BiFC interaction analysis, V33E-TIR1 failed both to detectably bind ASK1 (**S3F Fig**) and oligomerize (**S3E Fig**). However, the V33A/K35A-TIR1 variant retained a partial capacity to interact with ASK1 (**S3D Fig**) as well as the ability to oligomerize (**S3C Fig**).

To investigate further the role of ASK1 in TIR1 oligomerization, we performed a set of BiFC studies where TIR1 was co-expressed with ASK1. The results revealed an enhanced TIR1-TIR1 fluorescence signal when co-expressed with ASK1 (**S3G–S3I Fig)**. As suggested by others, SKP1 proteins can stabilize the conformation of F-box proteins most likely via Skp1-mediated masking of the hydrophobic F-box domain [32]. To assess whether enhanced TIR1 oligomerization in the presence of ASK1 was due to TIR1 stabilization, immunoblot analyses were performed in the presence of cycloheximide (a *de novo* protein synthesis inhibitor), which revealed that steady-state TIR1 protein levels were elevated in the presence of ASK1 (**S3N Fig**). To investigate the stability and localization of the mutant proteins employed in this study YFP-tagged mutant variants were generated, expressed in the Nicotiana expression system, and the localization and signal intensity of YFP tagged mutants were compared to that of YFP:TIR1 (**S3J Fig**). The V33A/K35A-TIR1 and V33E-TIR1 mutant proteins retained their nuclear localization (**S3K and S3L Fig),** although the fluorescence signals associated with these interactions were markedly reduced, suggesting a corresponding reduction in protein abundance. On the other hand, the fluorescence signal arising from YFP:TIR1-ΔF was confined to intense signal foci external to the nucleus, reminiscent of protein aggregation (**S3M Fig**), suggesting that the integrity of the F-box domain is essential for maintenance of TIR1 structure.

The results provided here do not conclusively rule out a contribution by ASK1 to TIR1 oligomerization. However, they suggest that the ASK1-mediated effect on TIR1 oligomerization is likely due to enhanced stability of TIR1 arising from ASK1 binding and masking of the hydrophobic F-box domain of TIR1, rather than a direct involvement of ASK1 in TIR1 oligomerization.

### A set of spatially-clustered amino acids are critical for TIR1 oligomerization

**Fig 3:**
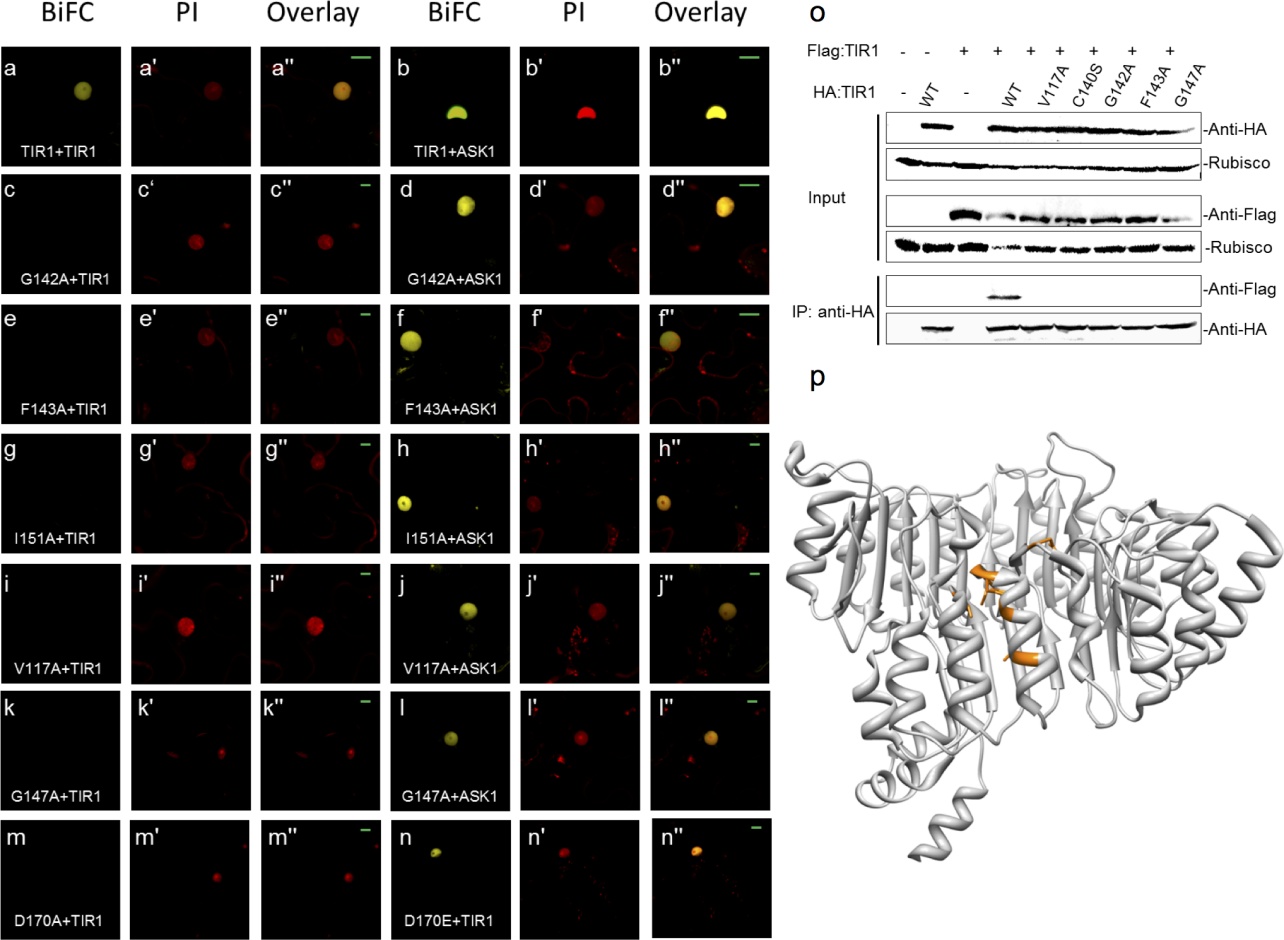
A set of spatially-clustered amino acids are critical for TIR1 oligomerization. **(a, c, e, g, i** and **k)** BiFC-based assessment of TIR1 oligomerization using wild-type and G142A, F143A, I151A, V117A and G147A mutants, respectively, following transient expression in Nicotiana leaves. **(b, d, f, h, j** and **l)** Assessment of TIR1-ASK1 interaction using wild-type and G142A, F143A, I151A, V117A and G147A mutants respectively, following transient expression in Nicotiana leaves. **(m** and **n)** Assessment of TIR1 interaction with D170A and D170E mutants using BiFC. **(o)** Co-IP experiments in Nicotiana leaves. HA-tagged wild-type and mutant Flag tagged TIR1 constructs were co-injected. Protein extracts were subjected to immuno-precipitation using anti-HA conjugated beads. The immune-precipitates were examined by western-blotting using anti-Flag and anti-HA antibodies. **(p)** Side view of TIR1-IAA7 peptide structure (PDB 2P1Q); residues critical for TIR1 oligomerization are depicted in orange.

In order to characterize the domain(s) responsible for dimerization, a set of *tir1* deletion mutants were generated and assessed for their ability to oligomerize. The results revealed that all deletion variants were highly unstable in a manner reminiscent of TIR1-ΔF. Thus, to identify potential residues critical for TIR1 oligomerization, we used both BiFC and Y2H approaches to assess the oligomerization ability of the jasmonate receptor COI1, a closely-related member of the TIR1-like AFB family (**S4C Fig**) [33]. Results from both approaches indicated that COI1 was able to oligomerize in a manner similar to that of TIR1 (**S4A and S4B Fig**). These results suggest that amino acids mediating oligomerization within this gene family are conserved between TIR1 and COI1 (**S5 Fig).** A total of 45 evolutionarily-conserved residues (highlighted in yellow **S5 Fig**) residing close to the surface of TIR1 and COI1 were mutated via an alanine scanning approach, and these variants were screened for their ability to oligomerize via BiFC. All but one of these mutants retained their ability to oligomerize. The lone mutant from this first screen found to have lost its ability to oligomerize was F143A-TIR1. This mutant, while unable to oligomerize nevertheless retained the ability to interact with ASK1 (**Fig 3**). To identify additional residues that functionally important for TIR1 oligomerization, conserved residues surrounding F143 were mutated. Among the missense mutants generated, several additional TIR1 variants that retained the ability to interact with ASK1, but lost the ability to oligomerize were identified, including V117A, G142A and I151A (**Fig 3**).

The *TIR1* locus was initially identified in forward genetic screens and implicated in auxin signaling upon the isolation and characterization of the *tir1-1* mutant allele carrying a single amino acid substitution of Gly147 to Asp (*tir1-1*) [17]. This mutation lies in close spatial proximity to amino acid residues characterized in our studies as critical for TIR1 oligomerization. To assess whether G147 also contributes to TIR1 oligomerization, we generated a Gly147A-*tir1* mutant allele and assessed its capacity to oligomerize. The results showed that the G147A variant lost its ability to oligomerize (**Fig 3**), but retained its ability to interact with ASK1. Interestingly, mutating the two residues immediately adjacent to G147 (D146 and L148) to Ala did not affect TIR1 oligomerization, suggesting that G147 is essential for oligomerization.

A recent study used forward genetic screens to identify two TIR1 mutations (D170E and M473L) that increase TIR1 interaction with Aux/IAAs [16]. When mutated to Ala at these positions, both mutant variants exhibited a decrease or complete loss of detectable Aux/IAA interaction. Strikingly, neither of these residues reside at the TIR1-Aux/IAA degron interaction interface, thus it is not clear how these residues impact Aux/IAA binding. Since the D170 residue spatially clusters with amino acids identified to be critical for oligomerization as described here, we assessed the competency of this allele for TIR1 oligomerization. While D170A-TIR1 lost the ability to oligomerize, the D170E mutant retained that same capacity (**Fig 3**). Co-IP analyses were employed to validate the loss or retention of oligomerization ability among the several TIR1 variants following transient expression *in vivo* (**Fig 3O**).

It may be important to note that all amino acid residues identified as critical for TIR1 oligomerization were found to spatially cluster in the TIR1 structure (**Fig 3P** and **S6 Fig**). Whereas all amino acid residues critical for oligomerization were found to reside close to surface of TIR1, the buried nature of their side chains suggests that leucine-rich repeats (LRR) 3, 4, and 5 represent the general domains involved in oligomerization of TIR1.

A recent study has provided evidence for NO-mediated modulation of auxin signaling through *S*-nitrosylation of TIR1 [13]. This redox-based post-translational modification of cysteine residues was found to enhance the interaction between TIR1 and Aux/IAA proteins via an unknown mechanism, even in the absence of auxin. Two critical residues, C140 and C480, have been postulated to act as the *S*-nitrosylation sites on TIR1 [34]. The mutational analyses included in these studies showed that a C140A-TIR1 variant protein lost the capacity for auxin-induced interaction with Aux/IAA proteins, whereas the C480A substitution severely reduced this interaction. Strikingly, neither of these two residues reside at the TIR1-Aux/IAA interaction interface. However, the close proximity of C140 to residues identified to be essential for oligomerization led us to investigate whether C140 can also contribute to TIR1 oligomerization. The results showed that C140A-TIR1 mutation reduced TIR1 oligomerization (**S7 Fig**) but retained the ability to interact with ASK1 (**S7 Fig)**. To verify that the inability of C140A-tir1 to oligomerize was not due to folding defects arising from C140 substitution to Ala, C140 was also mutated to Serine to generate C140S-tir1. This mutant also failed to oligomerize (**S7 Fig**) but retained the ability to interact with ASK1 in a manner similar to C140A-tir1 (**S7 Fig**). The inability of C140S-tir1 to oligomerize was also validated using Co-IP (**Fig 3O**). As part of a domain structure-function study, the two residues immediately adjacent to C140 (Ser139 and Glu141) were also mutated to assess their contribution to TIR1’s oligomerization; both variants retained their ability to oligomerize as revealed by BiFC studies.

Since all TIR1 oligomerization-deficient mutants retained the ability to interact with ASK1 in a manner similar to that of wild-type TIR1 (**Fig 3**), we concluded that differential interaction signals were not due to altered protein stability/structure profiles across the panel of TIR1 variants tested. To substantiate this conclusion, we measured the protein abundance of several of the mutant variants, following expression in the Nicotiana system using a cycloheximide chase assay. All protein variants analyzed exhibited similar stability profiles (**S8A and S8B Fig**), suggesting that the oligomerization phenotypes observed among the variants tested were not due to significant changes in protein stability.

### TIR1 oligomerization contributes to Aux/IAA7 binding and degradation

**Fig 4:**
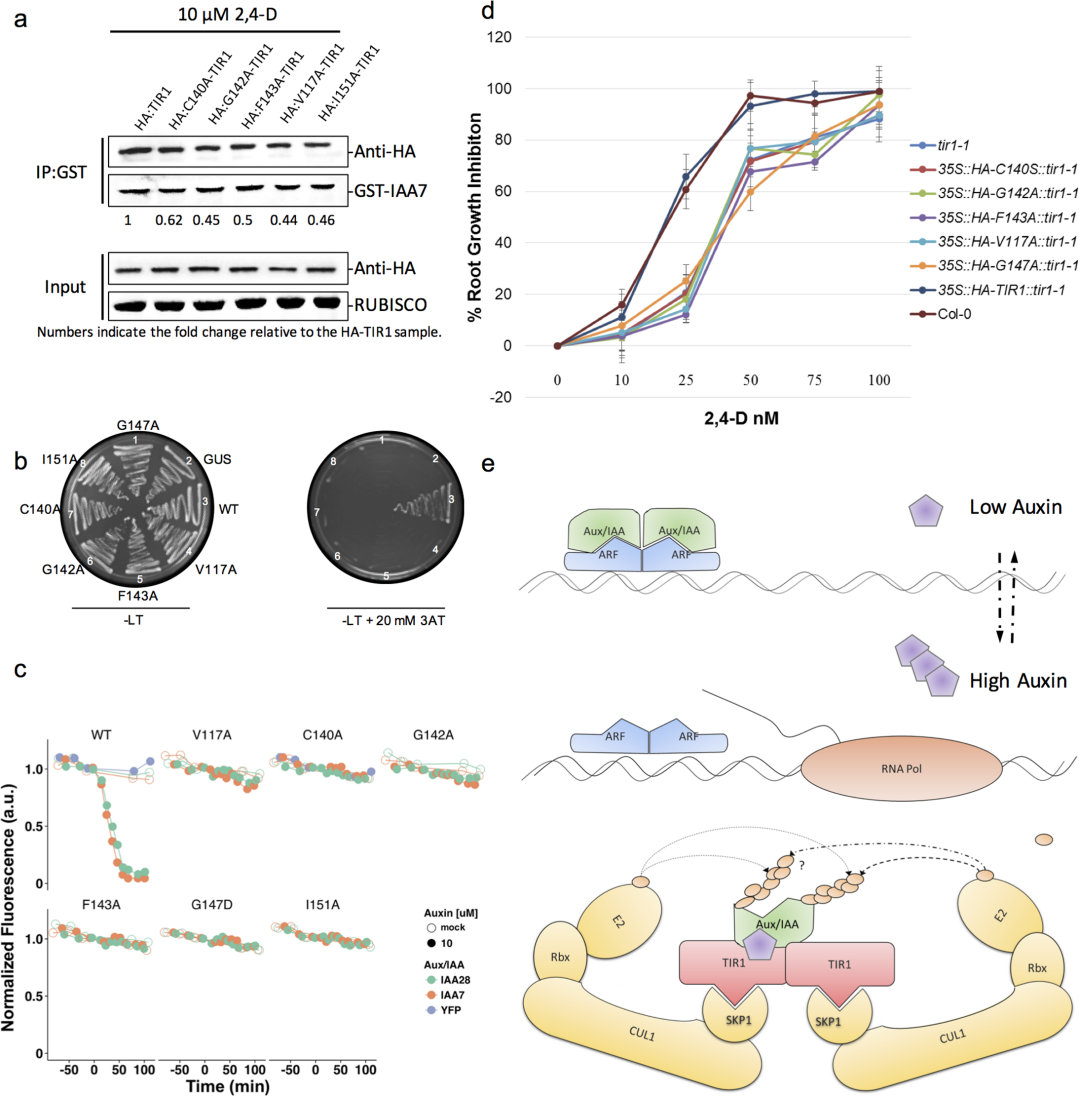
TIR1 oligomerization is essential for auxin signaling. **(a)** Semi *in vitro* pull-down assays using GST-IAA3 and Nicotiana cell extracts expressing wild-type and mutant HA:TIR1 proteins. Values represent fold-change compared to TIR1:HA samples (all values were corrected for equal loading among samples). **(b)** Assessment of TIR1-IAA7 protein interaction in Y2H assay in the presence of auxin (25 μM). Images of yeast cells expressing the designated constructs and grown on histidine plates and test plates containing 10 mM of 3-AT. **(c)** Degradation of YFP-IAA fusion proteins in yeast in the presence of wild-type and mutant TIR1 proteins after the addition of auxin. Yeast cells were imaged using time-lapse flow cytometry. Degradation curves were normalized to the starting fluorescence absorbance unit (a.u.). **(d)** Five day old seedlings were transferred to 0.5x MS mediums containing the indicated concentrations of 2,4-D. After 5 days of growth, the length of primary root was measured, and expressed relative to growth on control plates. **(e)** Schematic model of SCF^TIR1^ dimerization.

To assess whether TIR1 oligomerization is functionally significant, we assessed the binding behavior of TIR1 variants towards substrate proteins via affinity pull-down assays involving Nicotiana extracts expressing HA-tagged wild-type and mutant TIR1 protein variants. The results of these affinity-enrichment experiments revealed that GST-IAA3 preferentially co-enriches with wild-type versus mutant TIR1 proteins in the presence of auxin (**Fig 4A**). A Y2H assay revealed a similar pattern whereby various TIR1 mutants were unable to interact with IAA7 (**Fig 4B**). The data from both approaches suggests that TIR1 alleles competent for oligomerization preferentially interact with Aux/IAA substrates. In light of recent studies where additional residues outside of the Aux/IAA degron motif were suggested to contribute to their interaction with TIR1/AFBs [4], we hypothesize that oligomerization of TIR1 serves to enhance Aux/IAA protein interactions by engaging additional residues outside of the characterized degron motif. A similar mechanism has been recently reported for the human SCF^Fbw7^ complex, where only the dimeric form of Fbw7 was found to interact with the multiple degrons found in cyclin E [22].

To assess the effect of TIR1 oligomerization on substrate turnover, Myc:IAA3 was co-expressed with wild-type or mutant TIR1 variants *in planta*. Myc:IAA3 was significantly depleted when co-expressed with HA:TIR1 whereas IAA3 levels were elevated when co-expressed with TIR1 oligomerization-deficient mutants (**S9A Fig**). To extend upon these findings, and to eliminate the possible contribution of endogenous Nicotiana wild-type TIR1/AFB proteins on Aux/IAA protein degradation, we assessed the effect of TIR1 oligomerization on Aux/IAA degradation in yeast [15]. The conserved nature of the SCF Ub-ligase machinery in eukaryotes allows for the exploitation of yeast for ubiquitination and subsequent degradation of Aux/IAA proteins. To this end, the stability of YFP-tagged IAA28 and IAA7 fusion proteins were assessed using flow cytometry when co-expressed with wild-type and mutant TIR1 (**Fig 4C)**. While both IAA28 and IAA7 proteins were degraded by the wild-type SCF^TIR1^ complex upon auxin treatment in yeast, none of the mutants were competent to degrade either of the substrate proteins. Given that both Aux/IAA proteins (IAA7 and IAA28) exhibit differential degradation rates and binding affinities to TIR1 [4, 35], the inability of the TIR1 mutants to degrade the two substrates suggests that TIR1 oligomerization does not discern amongst various Aux/IAA proteins. To evaluate the functional importance of TIR1 oligomerization to auxin signaling *in planta*, transgenic Arabidopsis plants expressing the oligomerization-deficient *tir1* alleles V117A, C140S, G142A, G147A, and F143A were generated in a mutant *tir1-1* genetic background. Transgenic lines expressing similar levels of TIR1/tir1 protein (**S9B Fig**) were subjected to phenotypic analysis, including the induction of lateral roots and root elongation in five-day-old seedlings transferred onto 0.5x MS medium containing various concentrations of the synthetic auxin 2,4-D. While *tir1-1* mutant plants are deficient in auxin-induced lateral root formation and resistant to auxin-inhibitory effects on primary root elongation, ectopic expression of the wild-type TIR1 protein in the *tir1-1* background rescued both these phenotypes (**Fig 4D** and **S9C Fig**). In contrast, expression of the oligomerization mutants (C140A, G142A, F143A, V117A, and G147A) failed to rescue both of the auxin-response phenotypes observed in the *tir1-1* mutant background. Taken together, the data suggest that TIR1 oligomerization is an essential aspect of Aux/IAA binding and degradation, as well as downstream auxin signaling and perception in Arabidopsis.

## Discussion

Although significant advances have been made in our understanding of auxin perception in plants, the regulatory potential for auxin perception at the level of substrate interaction remains to be elucidated. One of the main questions in auxin perception is how this phytohormone can play such diverse roles during plant growth and development [1, 3]. Recent studies have reported variation in binding affinity among the AFB/TIR1 and Aux/IAA co-receptor protein pairs [10]. Furthermore, the differential binding profiles observed between TIR1 and various ASK proteins could significantly increase the combinatorial diversity and dynamic range of ASK-TIR1/AFB-Aux/IAA complex formation [36]. In the study presented here, we provide evidence that ASK1 co-expression is important for TIR1 stability. One implication of this observation, combined with our previous results showing that TIR1 and related proteins exhibit differential interaction profiles with the 21-member ASK protein family in Arabidopsis [36], suggest that various TIR1-ASK complexes with differing stability levels likely reside simultaneously within the proteome. An examination of the ASK1-TIR1 crystal structure reveals the most C-terminal α-helix of ASK1 protein is located in close proximity to the auxin-InsP_6_ binding site of TIR1. Hence, the stability of different TIR1/AFB-ASK complexes might manifest differential functional outcomes in auxin perception and ubiquitination of target Aux/IAAs.

Using multiple approaches, we provide evidence for the formation of higher-order SCF^TIR1^ structures via a novel dimerization domain, suggesting that domains other than the canonical D-domain can mediate F-box dimerization. The structure-function studies described here have identified a set of spatially clustered residues that are critical for TIR1 oligomerization. We also show that two residues previously shown to be critical in auxin signaling and response (G147 and D170) are coincidentally deficient for TIR1 oligomerization, strongly suggesting a role for TIR1 oligomerization in plant auxin signaling. As well, the conserved nature of the residues identified in TIR1 across the LRR class of F-Box proteins has broader implications for the characterization of other novel domains mediating dimerization of these F-box proteins in other eukaryotes.

While the functional significance of higher-order SCF structures are generally unknown, the biochemical and genetic approaches outlined here provide evidence for TIR1 oligomerization as a factor in both the regulation of Aux/IAA interaction and subsequent degradation (**Fig 4E**). The differential affinities observed among the various TIR1/AFB-Aux/IAA protein pairs provides a mechanism that can explain, in part, the deferential degradation rates observed of Aux/IAA proteins [4]. The positional context and stoichiometry of ubiquitin conjugation sites could also be a major regulator of SCF ligase substrates degradation rates [37, 38]. Since multiple lysine residues have been shown to be essential for degradation of several target Aux/IAA proteins, it seems likely that the degradation rate of select Aux/IAA proteins is also influenced by the context and number of lysine residues available to be ubiquitinated. The ubiquitination of multiple dispersed lysine residues via a monomeric SCF^TIR1/AFB^ ligase would be challenging however, the quaternary structure diversity afforded by oligomerization could provide a framework in which both the pattern and stoichiometry of Ub conjugation sites could be investigated.

Although Aux/IAA proteins can associate with SCF^AFB/TIR1^ through the degron sequence found within their domain II, the models currently proposed do not account for the size and sequence diversity of the 29-member Aux/IAA protein family. Given the sequence diversity of Aux/IAA proteins, it is conceptually challenging how a monomeric SCF^TIR1/AFB^ complex to accommodate and efficiently ubiquitinate diverse substrate proteins. Nonetheless, the distinct distances observed between the E2 binding site and substrate proteins following oligomerization, as observed in the case of SCF^CDC4^ dimerization [20], could provide a structural context in which the structurally diverse Aux/IAA proteins could interact and be efficiently ubiquitinated.

While *S*-nitrosylation was previously shown to enhance the ability of TIR1 to bind Aux/IAA in the absence of auxin, a molecular mechanism underlying this observation has not yet emerged [13]. Our findings in this study, in which amino acid residues believed to be important for *S*-nitroslylation also abolish TIR1 oligomerization, raises the question whether NO-mediated effects on auxin signaling are likely modulated via TIR1 oligomerization. Characterization of the precise residues that are *S*-nitrosylated on TIR1 could further our understanding of the role of redox based modification on auxin signaling.

Our study raises a number of questions, including whether the formation of distinct SCF^TIR1^ complexes involving different ASK proteins results in the differential regulation of Aux/IAA protein binding and/or degradation. It is also not clear whether TIR1 can form hetero-oligomers with other AFB proteins, with the potential of enhancing the structure-function diversity of SCF^TIR1^ ligases formed. Further, a role for *S*-nitrosylation in the regulation of TIR1 oligomerization, including the possibility that other redox-based modifications triggered by mechanisms unrelated to auxin signaling (e.g. innate immunity responses) might influence SCF^TIR1^ quaternary structure, thereby providing a novel regulatory perspective for the co-regulation of plant patterning with environmental inputs. The experimental data shown here provides a foundation for the investigation of these and related questions important for understanding the functional contribution of SCF^TIR1^ oligomerization to auxin signaling in plants, with broader implications for SCF structure function studies in other eukaryotes.

## Materials and Methods

### *N*. *benthamiana* transient expression system

Protein expression studies were performed in the *Nicotiana benthamiana* transient expression system as previously described [36]. For nuclear staining, leaves were injected 30–90 min prior to imaging with 10μg/ml of Propidium Iodide (PI) and kept in the dark. For MG132 (10 μM) or CHX (100 μM) treatment, plants were injected with solution 4–5 hours prior to extract preparation or imaging.

### Site directed mutagenesis

Site directed mutagenesis was performed essentially as described in [39] using pENTR221:TIR1 as template. Amplification and fusion of DNA fragments was carried out using Phusion® High-Fidelity DNA Polymerase (Fermentas). All mutants we subsequently cloned using LR clonase II Enzyme mix (Invitrogen) either into BiFC (BiFP2 or BiFP3) or pEarleygate (201 and/or 202) vectors. The constructs generated in this study are listed in S1 Table.

### Protein extraction and western blotting

Western blotting experiments were done as described previously[2].

### Semi *in vitro* pull-down assays

The *in vitro* GST-Aux/IAA (*pDEST15*:*IAA3 and pDEST15*:*IAA7*) pull-down assay was done using Nicotiana protein extracts expressing HA tagged wild-type or mutant TIR1. 1 mg of protein extract was incubated with 5 μg of GST-Aux/IAA beads in the presence 10 μM 2,4-D at 4°C for 4–8 hrs. Samples were eluted using reduced glutathione (Sigma) and resolved by SDS-PAGE. Proteins were detected by anti-HA antibody (Roche).

### Nicotiana co-immunoprecipitation

Co-IP based experiment are done using, using Nicotiana protein extracts (extraction buffer; 50 mM Tris, pH 7.5, 150 mM NaCl, 100mM MgCl2, 10% glycerol, 0.1% Nonidet P-40, complete protease inhibitor [Roche], complete phosphatase inhibitor [Peirce] and 10 μM MG132) from leaves co-expressing HA tagged wild-type or mutant TIR1 and wild-type Flag tagged TIR1. Immunoprecipitation was performed using EZview™ Red Anti-HA Affinity Gel (Sigma). The precipitated proteins were separated by SDS–PAGE and immunoblotting was performed using anti-FLAG M2 monoclonal Abs (Sigma) or by anti-HA antibody (Roche).

### Confocal imaging

Imaging of BiFC signals *in planta* was performed as described previously [2]. Corrected total cell fluorescence (CTCF) was calculated as described using data acquired from ImageJ on about 200 randomly selected cells. For purposes of calculation CTCF was determined as Integrated Density subtracted for the product of area of the selected cell times its mean fluorescence background reading.

### Yeast two-hybrid analyses

Yeast two-hybrid (Y2H) assays were performed in yeast strain MaV203 using Invitrogen ProQuest expression vectors and product protocols. The open reading frame for each clone was recombined into both pDEST32 [DNA-binding (DB) domain] and pDEST22 [Activation-domain (AD) domain] Gateway-compatible vectors. To assess the effect of IAA3 on TIR1-TIR1 interaction, *IAA3* cDNA was cloned into pAG-416-GPD-ccdB containing a GPD constitutive promoter and *URA3* selection marker. TIR1-TIR1 or TIR1-IAA3 protein interaction was assessed in media lacking histidine and supplemented with the indicated concentrations of 3-amintotriazole (3-AT).

### Yeast YFP-IAA degradation assays

Yeast degradation assays were performed as described previously [40]. Briefly, yeast strains co-expressing stably-integrated YFP-tagged IAA proteins and TIR1 variants were prepared by transferring a freshly grown colony from YPD plates into SC media. Flow cytometry was used to estimate the cell density (in events μL^-1^) in cultures diluted so that cells were in log phase 16 hr later and for the duration of the experiment. All cultures were grown at 30°C with shaking. Pre-auxin measurements were taken to establish baseline expression followed by addition of auxin (10 μM indole-3-acetic acid) or mock treatment (95% [v/v] ethanol). Measurements were acquired at 10 min intervals for 120 min following auxin treatment, while mock-treated controls were measured every hour. At least two independent replicates were performed and all data were normalized by subtracting background auto-fluorescence and normalizing to pre-auxin fluorescence levels for each strain.

### *In vitro* ubiquitination reactions

8-12-week-old Nicotiana leaves were flash frozen, ground to a fine powder and resuspended in 1 mL lysis buffer [25 mM Tris-Cl (pH 7.5), 10 mM NaCl, 10 mM MgCl_2_, 4 mM PMSF, 5 mM DTT, 10 mM ATP] per 1 g leaf tissue. Lysates were cleared by centrifugation twice at 10,000 *g* for 10 min. 100 ng of GST:IAA3 was incubated with 100 μL of lysate (~150 μg protein) supplemented with 100 μM MG132 or DMSO at 22°C. Reactions were quenched with 1 volume of 2X Laemmli sample buffer with β-mercaptoethanol (Bio-Rad, Cat. No. 161–0737) at the indicated time points, then boiled and subjected to SDS-PAGE and Western blotting.

### Generation of transgenic plants

The *35S*::*HA*:*TIR1* wild-type and mutant constructs were transformed into *Agrobacterium tumefaciens* strain AGL1 by electroporation, and the presence of transgenes was confirmed by *in situ* PCR. Plant transformations were performed using the floral dip method using *tir1-1* mutant plants [41]. Seeds from treated plants were harvested, sterilized and stratified at 4°C for 2–3 days prior to plating and germination on solid 0.5x MS medium containing 50 μM D,L-phosphinothricin (a gift from Bayer Crop Science Canada) using a rapid selection procedure. Four to five independent transgenic plants were subsequently selected for each analysis. The double transgenic *pTIR1*:*TIR1–GUS* and *pTIR1*:*gTIR1–VENUS* Arabidopsis plants were generated through crossing of individual transgenic lines. Both single transgenic lines had been generated in *tir1-1* mutant lines and were shown to readily rescue the auxin mutant phenotype observed [26, 27]. F3 generation Arabidopsis plants were used for Co-IP based experiments.

## Supporting Information

S1 Fig Arabidopsis Aux/IAA protein abundance is regulated via SCF^TIR1^ E3 ligase in *N*. *benthamiana*. (**a**) Time course degradation of GST-IAA3 protein by viable extracts from 5-6-week-old Nicotiana plants in the presence and absence of MG132. The *in vitro* degradation assay suggests that GST-IAA3 protein abundance is regulated in an MG132-dependent manner. Multiple experimental repetitions yielded similar results. A semi-quantitative analysis of GST-IAA3 protein levels (relative to loading controls), derived from the western blot (c), is graphed. (**b**) Myc:IAA7 was expressed in Nicotiana leaves and was subjected to MG-132 (10 μM) or DMSO treatment for 5 h prior to protein extraction. IAA7 protein abundance was markedly increased following administration of MG132 (**c**) Myc:IAA7 and HA:TIR1 were transiently co-expressed in Nicotiana leaves and visualized using anti-Myc and anti-HA antibodies, respectively. The large subunit of Rubisco was used as a loading control. IAA7 protein abundance was markedly reduced when co-expressed with Arabidopsis TIR1; suggesting that TIR1 can assemble as part of a SCF ligase complex and subsequently target the Arabidopsis IAA7 protein for degradation via the 26S/proteasome system in Nicotiana.
(TIFF)

S2 Fig The effect of auxin and Insp6 binding on TIR1 oligomerization. (**a**-**d**) BiFC-based assessment of wild-type TIR1 interaction with R403A, L439A, S438A/L438A and K113A/R114A TIR1 mutants following transient expression in Nicotiana leaves. (**a'**-**d'**). Propidium iodide staining of the nucleus.
(TIFF)

S3 Fig The effect of ASK1 on TIR1 oligomerization. (**a**, **c** and **e**) BiFC-based assessment of TIR1 oligomerization using ΔF-TIR1, V33A/K35A-TIR1 and V33E-TIR1, respectively. (**b**, **d** and **f**) BiFC-based assessment of ASK1 binding with ΔF-TIR1, V33A/K35A-TIR1 and V33E-TIR1, respectively. (**g** and **h**) BiFC-based assessment of TIR1 oligomerization in the absence and presence of Myc:ASK1, respectively. Leaves were subjected to 100 μM of CHX treatment 5 hrs prior to imaging (**a'-h'**) Propidium iodide staining of the nucleus. (**i**) Corrected total cell fluorescence (n~50) ± SD of TIR1 oligomerization corresponding to figures (**g** and **h**). (**j**) Western blotting on protein cell extracts from Nicotiana leaves expressing either HA:TIR1 alone or co-expressing Myc:ASK1 and HA:TIR1. Leaves were treated with 100 μM CHX 5 hrs prior to protein extraction. (**k-n**) Assessment of sub-cellular localization of YFP-TIR1, YFP-V33A/K35A-TIR1,YFP-V33E-TIR1 and YFP-ΔF + TIR1, respectively.
(TIF)

S4 Fig Assessment of COI1-COI1 oligomerization. **(a)** BiFC-based assessment of COI1 oligomerization following transient expression in Nicotiana leaves. (**a')** Propidium iodide (PI) staining of the nucleus. (**b**) Assessment of COI1-COI1 protein interaction in Y2H assay. Images of single colonies expressing the designated constructs and grown on histidine plates (top panel) and test plates containing 10 mM of 3-AT without histidine (bottom panels). (**c)** The phylogenetic grouping of *TIR1*, *AFB1-5* and *COI1* genes based on their deduced primary amino acid sequence.
(TIF)

S5 Fig Alignment of Arabidopsis TIR1, AFB1-5 and COI1. Sequence alignment of *TIR1*, *AFB1-5* and *COI1* genes based on their deduced primary amino acid sequence. Conserved residues are highlighted in yellow.
(TIFF)

S6 Fig Amino acids essential for TIR1 oligomerization spatially cluster. Two views of the Superimposed SCF^TIR1^ complex structure are shown as a ribbon diagram. TIR1, ASK1, CUL1, RBX1, and the IAA7 substrate peptide are colored gray, dark blue, green, red and yellow, respectively. Amino acids critical for TIR1 oligomerization are colored in orange. Superimposing of ASK1-TIR1 (PDB 2P1Q) and SKP1-CUL1-RBX1 (1LDK) was performed using Swiss PDB Viewer.
(TIFF)

S7 Fig Cysteine 140 is essential for TIR1 oligomerization. **(a, c)** Visualization of BiFC-based TIR1 oligomerization between wildtype and C140A and C140S respectively in Nicotiana epidermal leaf cells. (**e and g)** Visualization of BiFC-based ASK1-TIR1 interaction between wild-type ASK1 and C140A- and C140S-TIR1 mutants in Nicotiana epidermal leaf cells.
(TIFF)

S8 Fig Wild-type and mutant TIR1 proteins exhibit similar stability profiles. **(a)** Wild-type and mutant HA:TIR1 protein decay levels assessed following and transient expression in Nicotiana using immunoblot and administration of 100 μM of CHX. **(b)** A semi-quantitative analysis of TIR1 protein levels (relative to loading controls), derived from the Western blots shown, are graphed.
(TIF)

S9 Fig TIR1 oligomerization is essential for Auxin Signaling. **(a)** Western blot analysis of protein cell extracts from Nicotiana leaves expressing either Myc:IAA3 alone or co-expressing Myc:IAA and wild-type or mutant HA:TIR1 **(b)** Western blot analysis of transgenic seedling Arabidopsis plants expressing various *TIR1/tir1* transgenes. Plants expressing similar protein levels were chosen for phenotypic analysis. **(c)** Five day old seedlings were transferred to fresh 0.5x MS (25 nM 2,4-D) mediums and lateral root formation was calculated.
(TIFF)

S1 Table The plasmid constructs generated in this study (XLSX)
