# Supplementary figures and images for: Oligomerization of SCFTIR1 Is Essential for Aux/IAA Degradation and Auxin Signaling in Arabidopsis
Source: PLoS Genet. 2016 Sep 12;12(9):e1006301. doi: 10.1371/journal.pgen.1006301 (PMC5019376; doi:10.1371/journal.pgen.1006301)

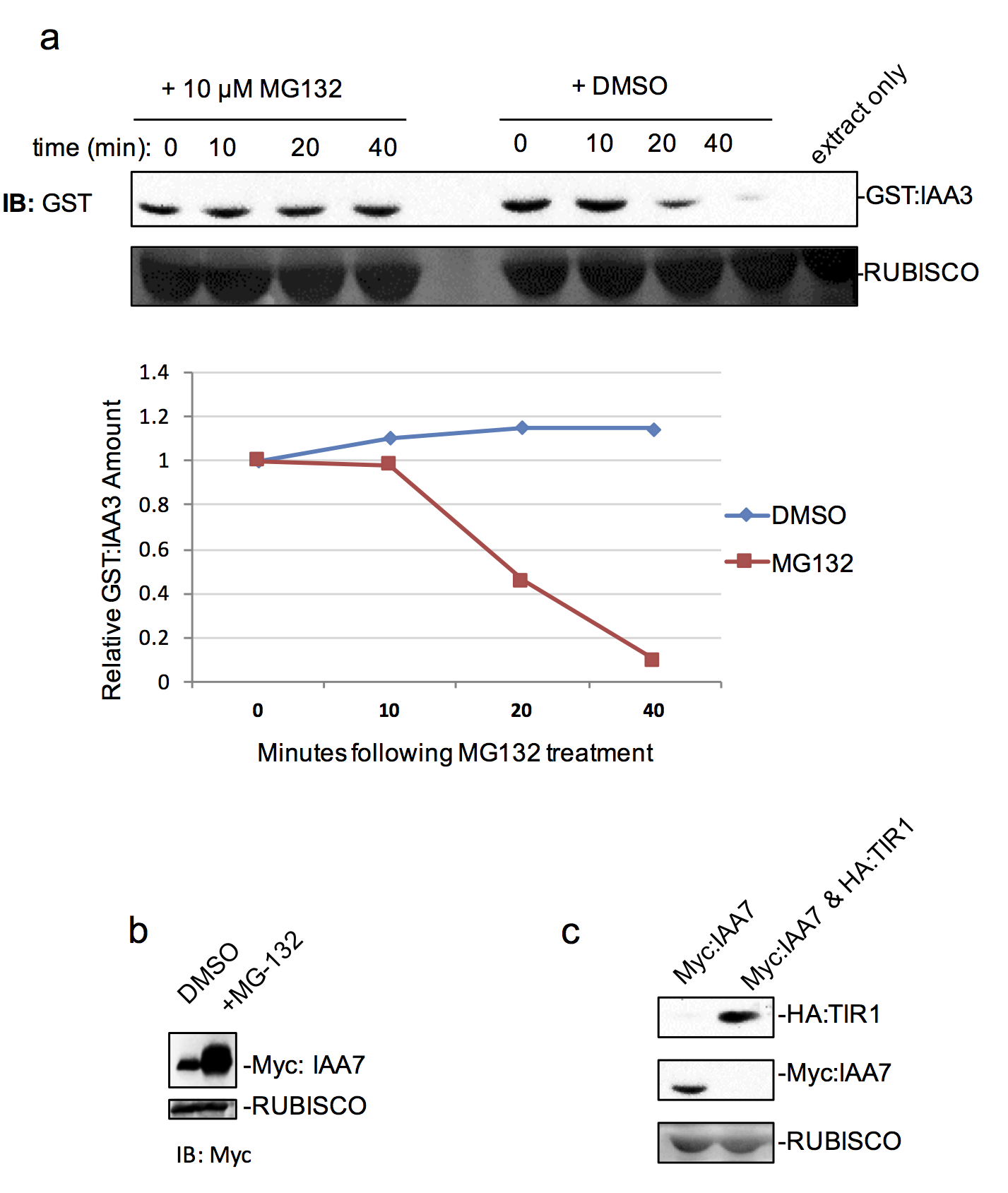

Supplement: S1 Fig — (a) Time course degradation of GST-IAA3 protein by viable extracts from 5-6-week-old Nicotiana plants in the presence and absence of MG132. The in vitro degradation assay suggests that GST-IAA3 protein abundance is regulated in an MG132-dependent manner. Multiple experimental repetitions yielded similar results. A semi-quantitative analysis of GST-IAA3 protein levels (relative to loading controls), derived from the western blot (c), is graphed. (b) Myc:IAA7 was expressed in Nicotiana leaves and was subjected to MG-132 (10 μM) or DMSO treatment for 5 h prior to protein extraction. IAA7 protein abundance was markedly increased following administration of MG132 (c) Myc:IAA7 and HA:TIR1 were transiently co-expressed in Nicotiana leaves and visualized using anti-Myc and anti-HA antibodies, respectively. The large subunit of Rubisco was used as a loading control. IAA7 protein abundance was markedly reduced when co-expressed with Arabidopsis TIR1; suggesting that TIR1 can assemble as part of a SCF ligase complex and subsequently target the Arabidopsis IAA7 protein for degradation via the 26S/proteasome system in Nicotiana. (TIFF) [file pgen.1006301.s001.tiff]

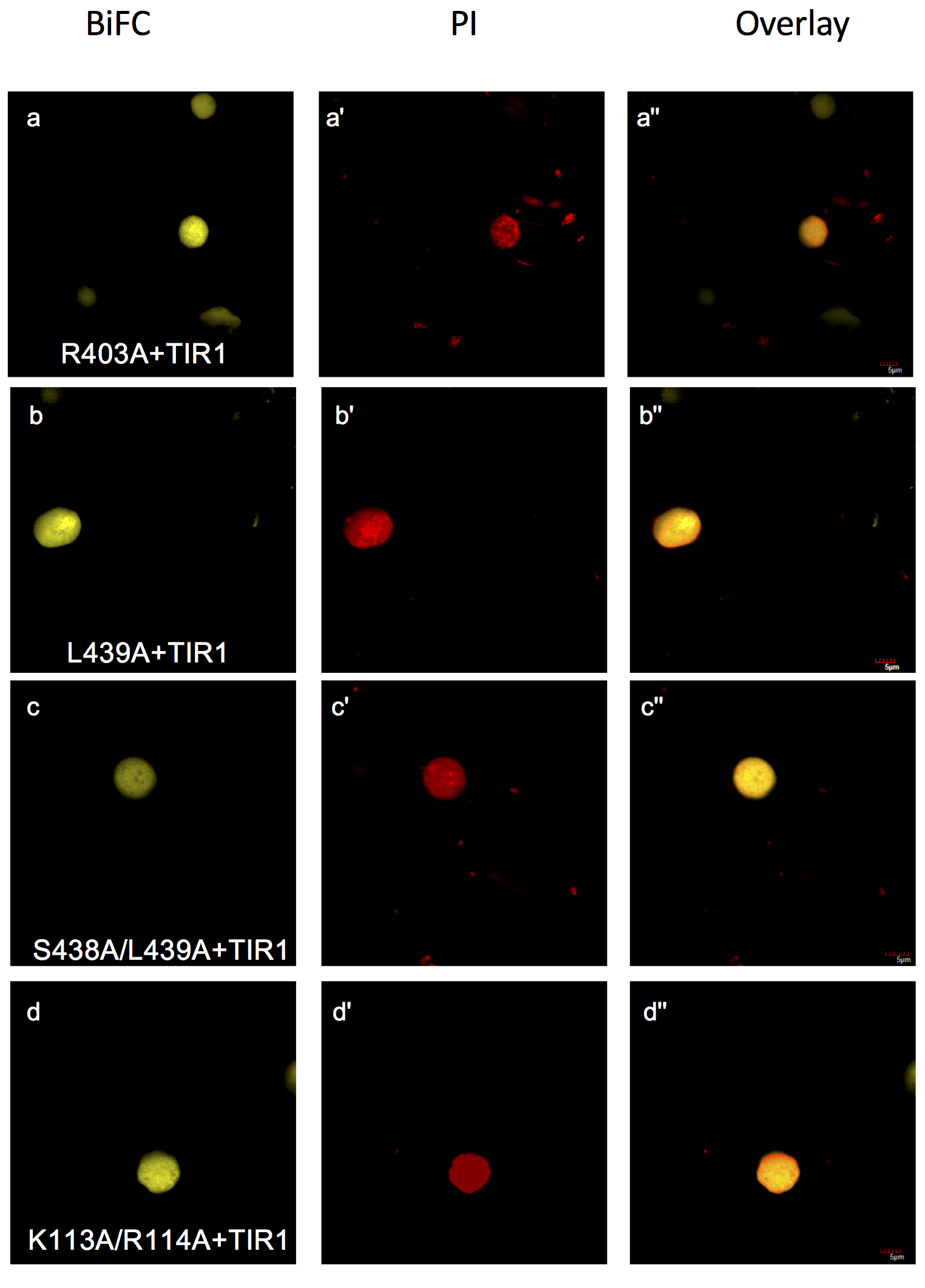

Supplement: S2 Fig — (a-d) BiFC-based assessment of wild-type TIR1 interaction with R403A, L439A, S438A/L438A and K113A/R114A TIR1 mutants following transient expression in Nicotiana leaves. (a'-d'). Propidium iodide staining of the nucleus. (TIFF) [file pgen.1006301.s002.tiff]

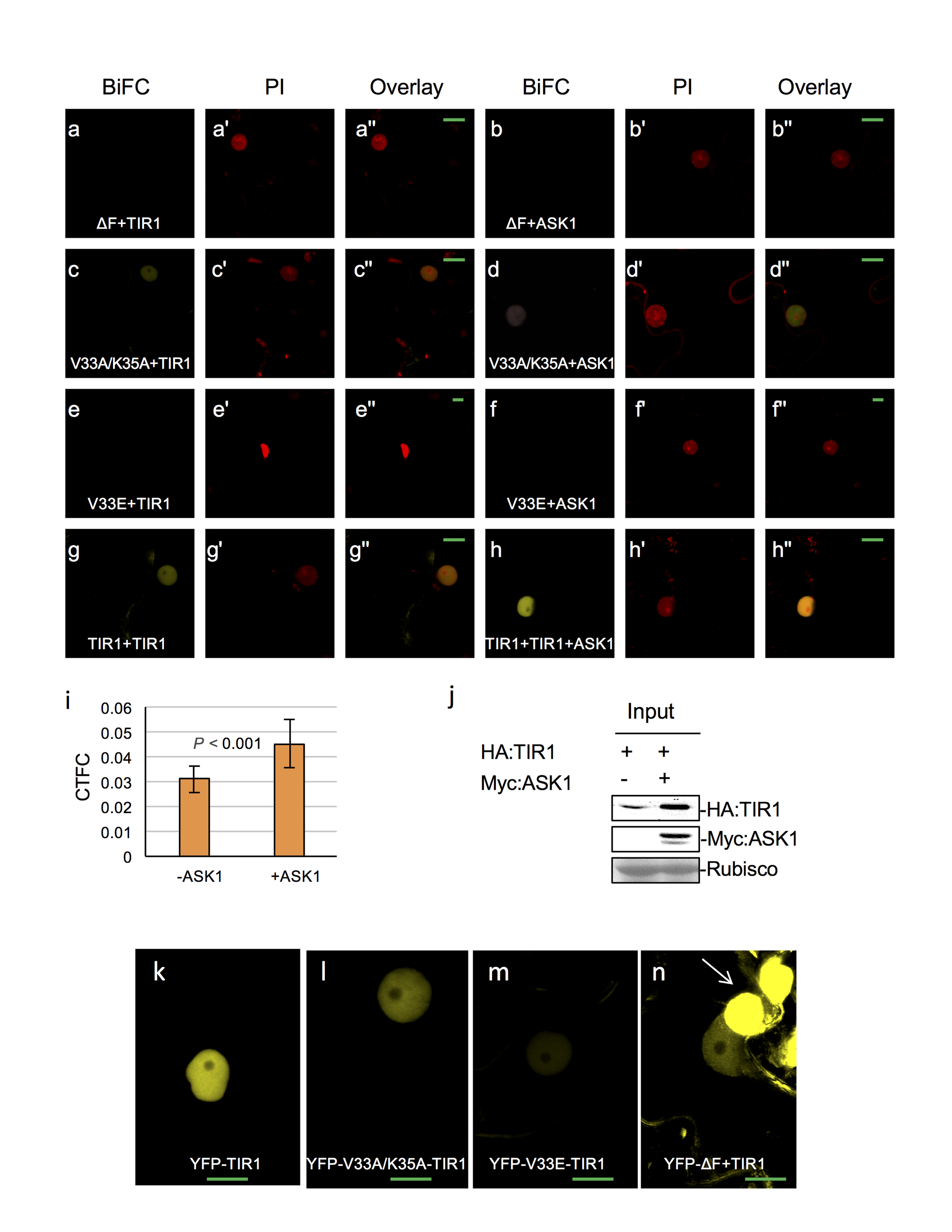

Supplement: S3 Fig — (a, c and e) BiFC-based assessment of TIR1 oligomerization using ΔF-TIR1, V33A/K35A-TIR1 and V33E-TIR1, respectively. (b, d and f) BiFC-based assessment of ASK1 binding with ΔF-TIR1, V33A/K35A-TIR1 and V33E-TIR1, respectively. (g and h) BiFC-based assessment of TIR1 oligomerization in the absence and presence of Myc:ASK1, respectively. Leaves were subjected to 100 μM of CHX treatment 5 hrs prior to imaging (a'-h') Propidium iodide staining of the nucleus. (i) Corrected total cell fluorescence (n~50) ± SD of TIR1 oligomerization corresponding to figures (g and h). (j) Western blotting on protein cell extracts from Nicotiana leaves expressing either HA:TIR1 alone or co-expressing Myc:ASK1 and HA:TIR1. Leaves were treated with 100 μM CHX 5 hrs prior to protein extraction. (k-n) Assessment of sub-cellular localization of YFP-TIR1, YFP-V33A/K35A-TIR1,YFP-V33E-TIR1 and YFP-ΔF + TIR1, respectively. (TIF) [file pgen.1006301.s003.tif]

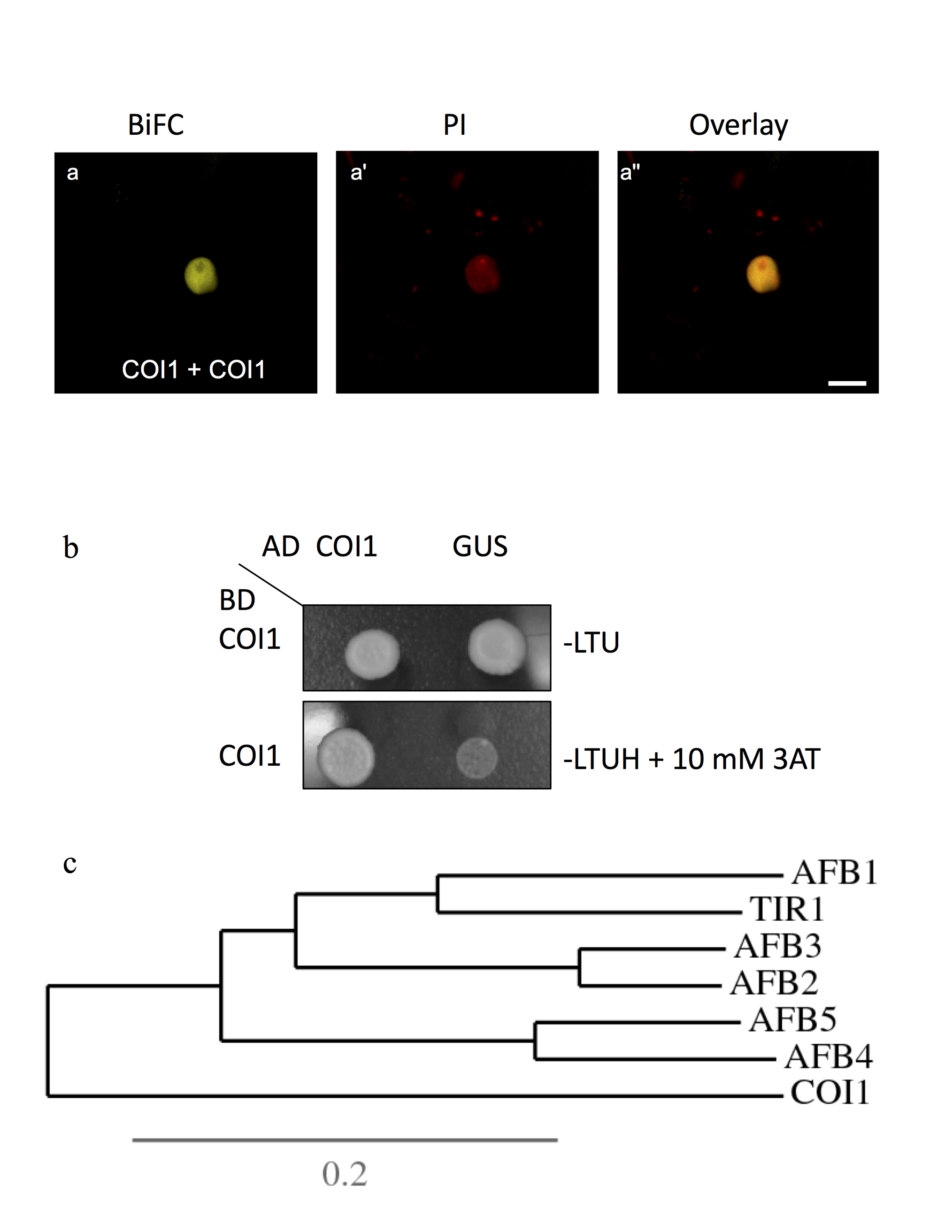

Supplement: S4 Fig — (a) BiFC-based assessment of COI1 oligomerization following transient expression in Nicotiana leaves. (a') Propidium iodide (PI) staining of the nucleus. (b) Assessment of COI1-COI1 protein interaction in Y2H assay. Images of single colonies expressing the designated constructs and grown on histidine plates (top panel) and test plates containing 10 mM of 3-AT without histidine (bottom panels). (c) The phylogenetic grouping of TIR1, AFB1-5 and COI1 genes based on their deduced primary amino acid sequence. (TIF) [file pgen.1006301.s004.tif]

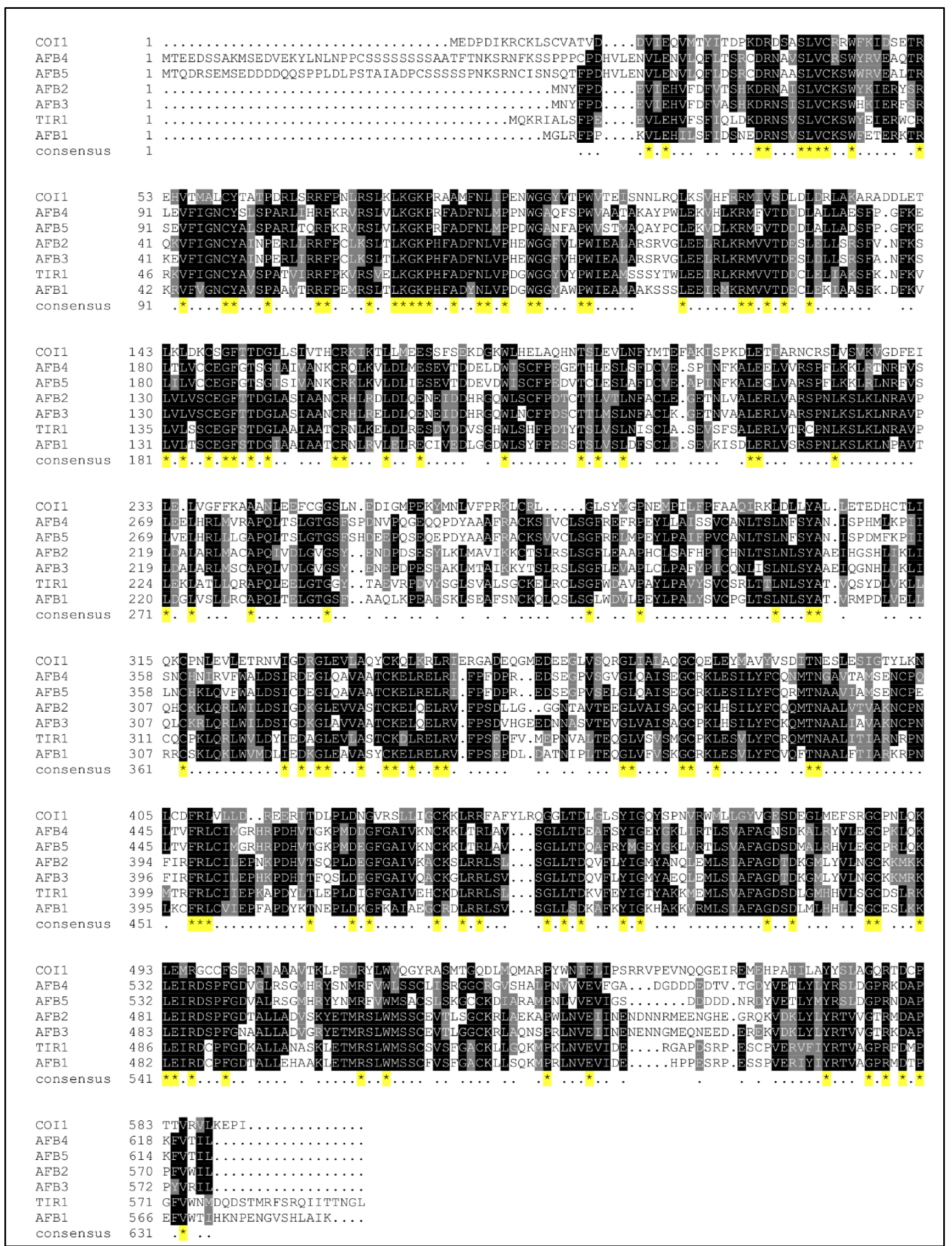

Supplement: S5 Fig — Sequence alignment of TIR1, AFB1-5 and COI1 genes based on their deduced primary amino acid sequence. Conserved residues are highlighted in yellow. (TIFF) [file pgen.1006301.s005.tiff]

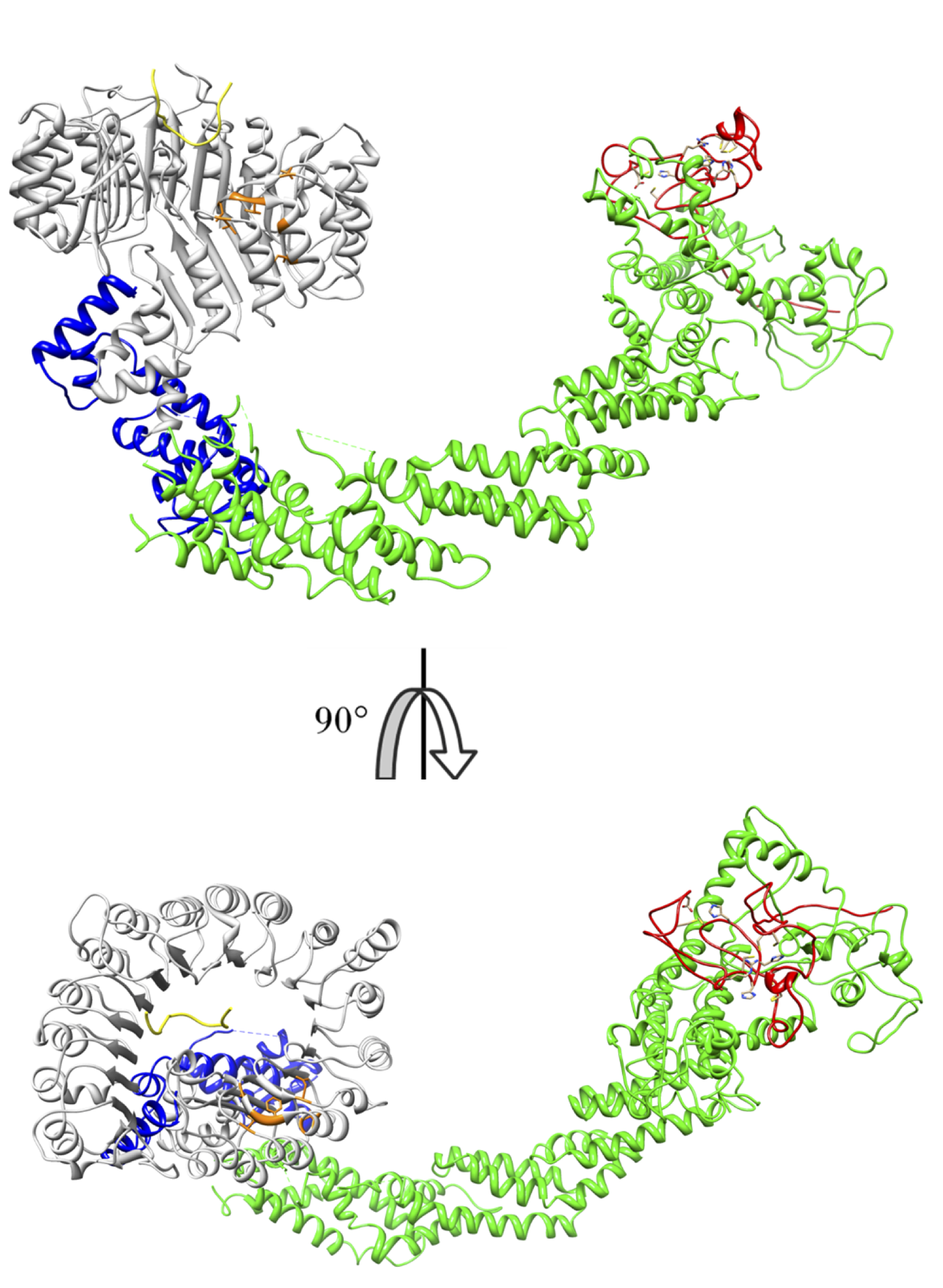

Supplement: S6 Fig — Two views of the Superimposed SCFTIR1 complex structure are shown as a ribbon diagram. TIR1, ASK1, CUL1, RBX1, and the IAA7 substrate peptide are colored gray, dark blue, green, red and yellow, respectively. Amino acids critical for TIR1 oligomerization are colored in orange. Superimposing of ASK1-TIR1 (PDB 2P1Q) and SKP1-CUL1-RBX1 (1LDK) was performed using Swiss PDB Viewer. (TIFF) [file pgen.1006301.s006.tiff]

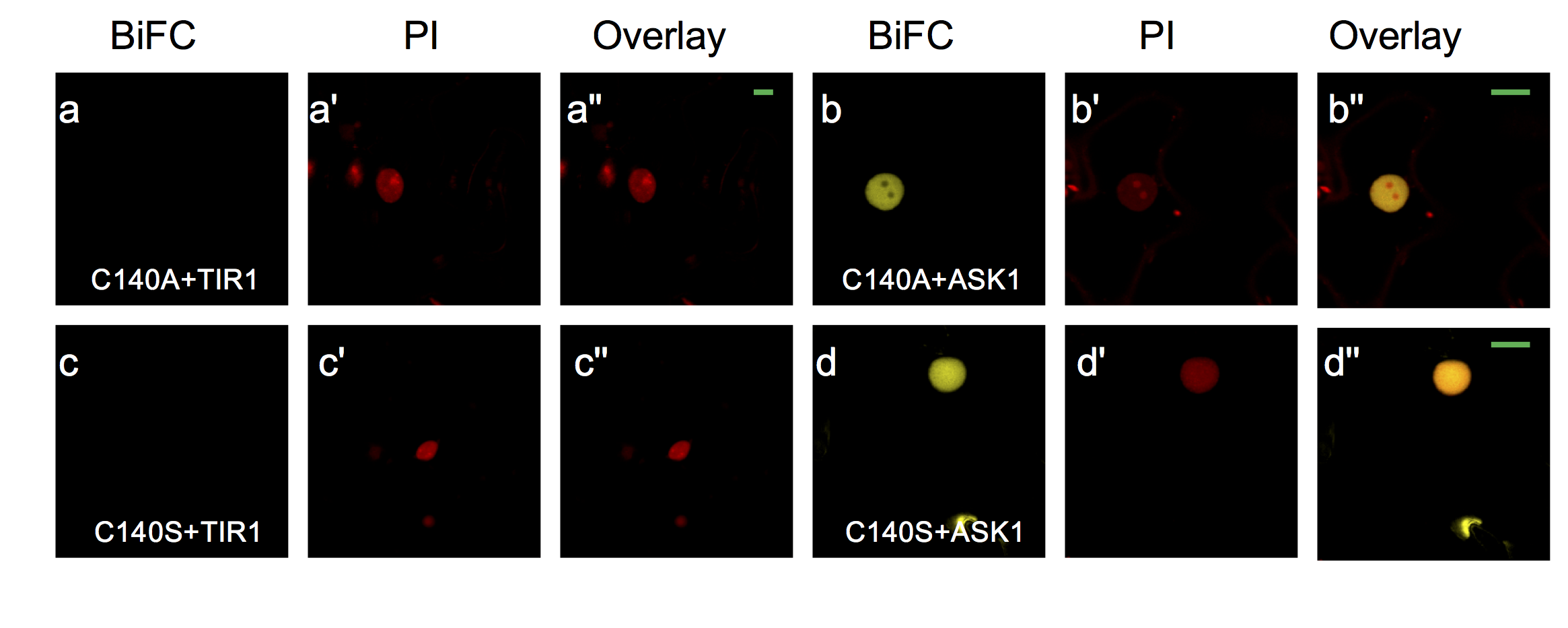

Supplement: S7 Fig — (a, c) Visualization of BiFC-based TIR1 oligomerization between wildtype and C140A and C140S respectively in Nicotiana epidermal leaf cells. (e and g) Visualization of BiFC-based ASK1-TIR1 interaction between wild-type ASK1 and C140A- and C140S-TIR1 mutants in Nicotiana epidermal leaf cells. (TIFF) [file pgen.1006301.s007.tiff]

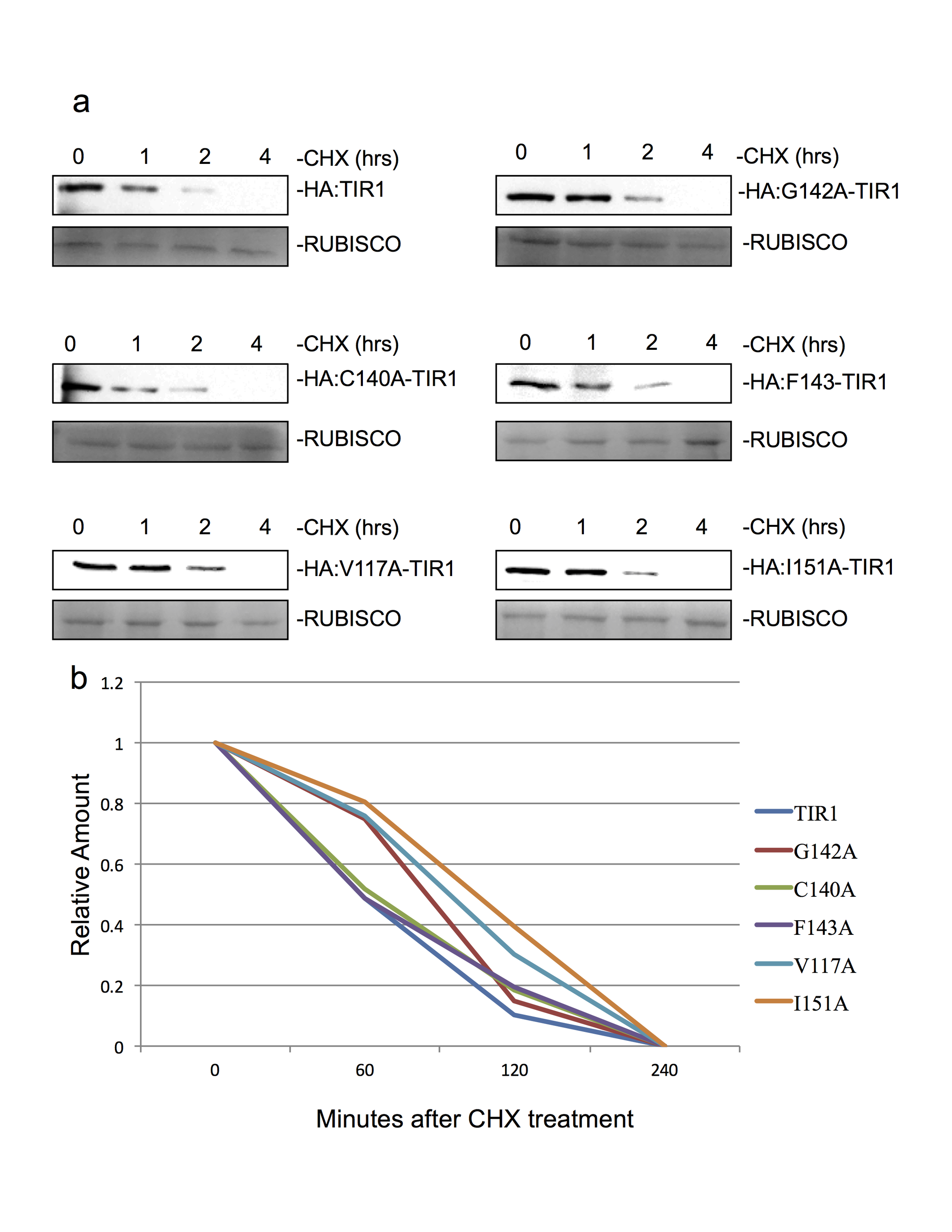

Supplement: S8 Fig — (a) Wild-type and mutant HA:TIR1 protein decay levels assessed following and transient expression in Nicotiana using immunoblot and administration of 100 μM of CHX. (b) A semi-quantitative analysis of TIR1 protein levels (relative to loading controls), derived from the Western blots shown, are graphed. (TIF) [file pgen.1006301.s008.tif]

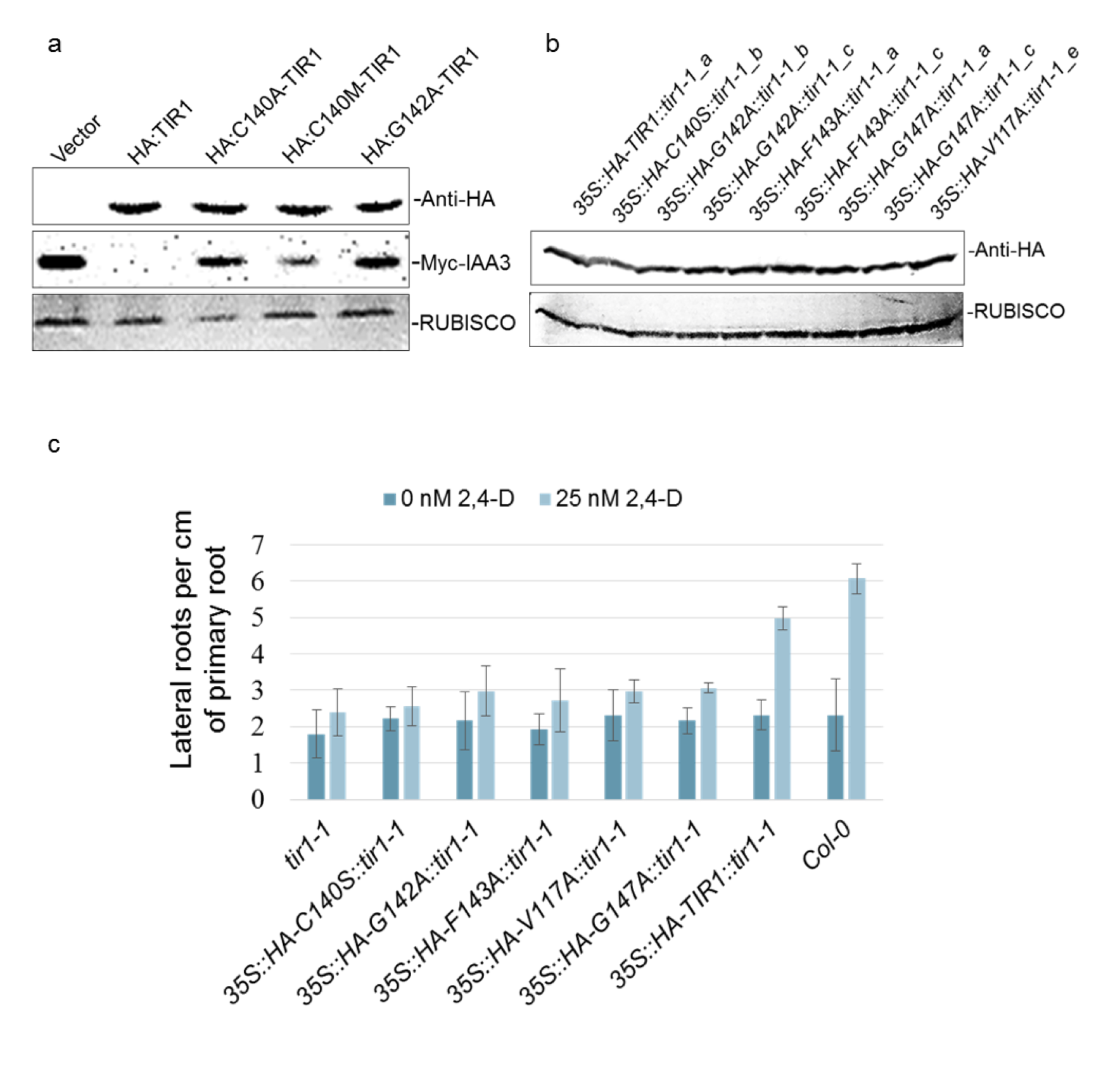

Supplement: S9 Fig — (a) Western blot analysis of protein cell extracts from Nicotiana leaves expressing either Myc:IAA3 alone or co-expressing Myc:IAA and wild-type or mutant HA:TIR1 (b) Western blot analysis of transgenic seedling Arabidopsis plants expressing various TIR1/tir1 transgenes. Plants expressing similar protein levels were chosen for phenotypic analysis. (c) Five day old seedlings were transferred to fresh 0.5x MS (25 nM 2,4-D) mediums and lateral root formation was calculated. (TIFF) [file pgen.1006301.s009.tiff]
